# Patient‐based experiences with the use of an ambulatory electromyographic device for the assessment of masticatory muscle activity during sleep

**DOI:** 10.1111/joor.12945

**Published:** 2020-03-06

**Authors:** Magdalini Thymi, Merel C. Verhoeff, Corine M. Visscher, Frank Lobbezoo

**Affiliations:** ^1^ Department of Orofacial Pain & Dysfunction Academic Centre for Dentistry Amsterdam (ACTA) University of Amsterdam Amsterdam The Netherlands

**Keywords:** ambulatory, electromyography, experience, mixed methods, sleep bruxism, smartphone

## Abstract

**Background:**

It is important to know how easy or difficult it is to use an ambulatory electromyographic (EMG) device for sleep bruxism assessment, and how this might affect its future utilisation.

**Objective:**

To explore the experience of individuals using an EMG device that pairs with a smartphone app, in order to detect factors that could facilitate and/or hamper its utilisation in future scientific research.

**Methods:**

Fifteen adults were recruited in the Orofacial Pain and Dysfunction Clinic of the Academic Centre for Dentistry Amsterdam (ACTA). Overnight recordings were performed in the home setting during one week. Time investment, feelings and thoughts, encountered difficulties and reasons for not using the device were assessed in a diary through open‐ended questions and 5‐point Likert scales. Content analysis of textual data was performed, and descriptives of quantitative data were calculated.

**Results:**

Time investment was low (mean 10.2 minutes in the clinic, and 1.9 minutes per recording at home). Quantitative data showed an overall good experience (median of 4). Qualitative diary data showed that the desire to gain insight into one's masticatory muscle activity formed the main motivation to use the device. Device detachment and difficulty in using the app were the most prominent negative experiences.

**Conclusion:**

The EMG device was well accepted for multiple overnight recordings. Curiosity for gaining insight into muscle activity was the most important factor that facilitated its use, and the app addressed this need. Device detachment and difficulties in using the app were the main factors that hampered its use.

## 1 BACKGROUND

Sleep bruxism is a masticatory muscle activity during sleep with rhythmic and non‐rhythmic features^1^ with potential negative oral health consequences, such as musculoskeletal symptoms, tooth wear and complications of restorative dental treatments.^2^ The activity occurs in most people,^2^ and, to some extent, its frequency and intensity vary over time.^3^ The development of an ideal assessment tool remains of high priority in the sleep bruxism research agenda.^1^ Patient self‐report and/or clinical examination are extensively used.^4^ These methods are simple, low cost and readily available, but unfortunately lack validity.^1^ Instrumental methods that provide electromyographic (EMG) data of masticatory muscle activity are currently suggested for accurate sleep bruxism assessments.^1^ Polysomnography (PSG), preferably with audio‐video (AV) recordings, has long been considered the gold standard for a definitive sleep bruxism diagnosis.^5^ PSG is a multiple‐channel sleep study, which requires set‐up, analysis, and interpretation by trained professionals.^6^ This procedure has substantial financial and feasibility implications, and makes multiple consecutive recordings a burdening task. Therefore, PSG is not suitable for the clinician seeking a simple diagnostic method for daily practice,^7^ and it poses significant challenges for the research setting in terms of study budgets and participant recruitment.

Portable EMG devices can produce masticatory EMG data and have the potential to overcome PSG‐related issues of feasibility and cost‐effectiveness.^8^ They can be self‐administered at the home setting, for single‐ or multiple‐night recordings.^8^ On the other hand, they may overestimate sleep bruxism activity, compared with PSG.^7^, ^9^ Validity of a diagnostic device is obviously one of its most important features, and studies on the validity of new diagnostic devices are crucial. However, even the most valid device will not be suitable for widespread use if it causes significant problems, or burden to the user, especially when it is aimed for use during sleep.^10^ The experience of burden can be affected by factors such as how easy or difficult it is to use the device, time spent mounting the device, complexity of handling the device's components and discomfort caused by attached device components and wires. It can be hypothesised that the small number, or even total absence of wires, and the possibility for self‐administration at home make the burden of portable EMG devices lower compared with PSG. However, this burden may still be significant, especially in the case of multiple‐night recordings. It may also be hypothesised that the burden arising from sleeping with a portable EMG device for several nights may affect the outcomes of sleep bruxism research, mainly by being a reason for participants to not fully adhere to study protocols.

Portable EMG devices have been used in sleep bruxism studies (eg 11, 12, 13, 14, 15, 16), and their further development may prove extremely useful for future research. To our knowledge, however, no studies have addressed the issue of how participants experience the use of such devices. Therefore, the aim of this study was to explore the experience of individuals with the use of a portable EMG device (BUTLER^®^ GrindCare^®^, Sunstar Suisse SA) for the assessment of masticatory muscle activity during sleep, in order to detect factors that could facilitate and/or hamper its utilisation in future scientific research.

## 2 METHODS

### 2.1 Study design

A mixed methods cohort study was designed, in which individuals’ experiences were explored by means of qualitative methods, that is, open‐ended questions collected through daily diaries and supported by descriptive quantitative data, that is, 5‐point Likert scales embedded in the same diaries. During 1 week, participants performed overnight EMG recordings in their home setting and completed the daily diary. Ethical approval was acquired by the local medical ethics committee (Medical Ethics Review Committee of VU University Medical Center, reference 2017.354).

### 2.2 Study population

Participants were recruited among the patients attending the clinic of Orofacial Pain and Dysfunction of the Academic Centre for Dentistry Amsterdam (ACTA). This clinic receives referrals from primary care related to temporomandibular dysfunction, oro‐facial pain, tooth wear, dental sleep disorders and bruxism. Inclusion criteria were as follows: age 18 years or older, diagnosis of probable sleep bruxism^5^ and sufficient understanding of the Dutch language in reading and writing. The following exclusion criteria were applied: patient categorised as class 3 or higher according to the American Society of Anesthesiologists (ASA) system for classification of physical status,^17^ presence of a pacemaker, known allergy to the EMG gel pad material, pregnancy, and presence of oro‐facial pain that is triggered by touch of the facial skin. Patients fulfilling these criteria were informed by the investigator about the study and were given a week time to consider participation. The investigator consulted with the clinician before approaching patients, to discuss whether study participation might interfere with regular care. The latter involves counselling and one or more of the following: physical therapy, psychological therapy, occlusal splint therapy, pharmacological treatment and, in cases of severe tooth wear, restorative treatment. When a patient was interested in participation, an appointment with the investigator was made, adjacent to the next regular clinic visit, during which informed consent was signed and the device was handed out after appropriate instructions on its use were given, as described below. An appointment was made for returning the device, for which participants were free to choose the location and time, in order to keep the burden of the study low. If regular care involved placement of a new occlusal splint, the device was given after the splint habituation period (ie after 2 weeks) as to avoid interference of any discomfort by the new splint with the study outcomes. Participants were asked to use the device for at least one night and were encouraged to use it for as many nights as possible, with a maximum of seven. Compensation for study‐related travelling costs was given, and participants received an oral hygiene “goodie bag” at the time of enrolment, which was provided to ACTA by Sunstar Suisse SA.

### 2.3 Electromyographic device

**Figure 1:**
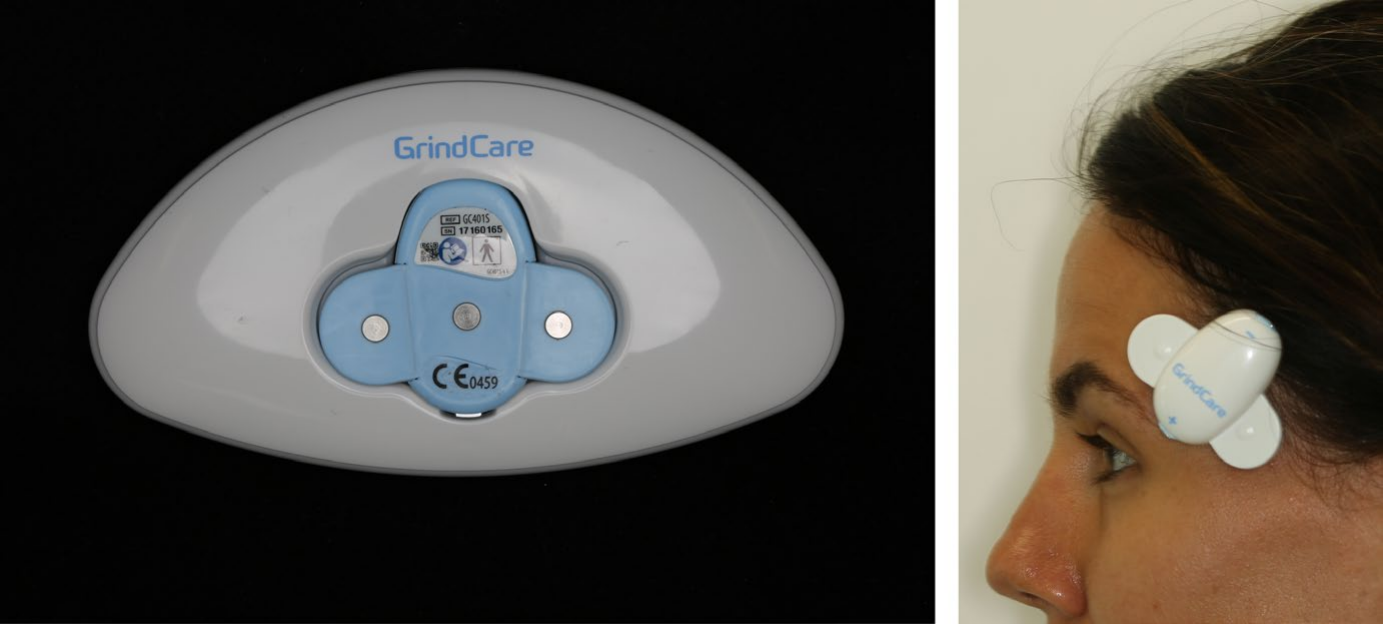
The BUTLER^®^ GrindCare^®^ device, left: the device in the docking station and right: the device attached to the skin

**Figure 2:**
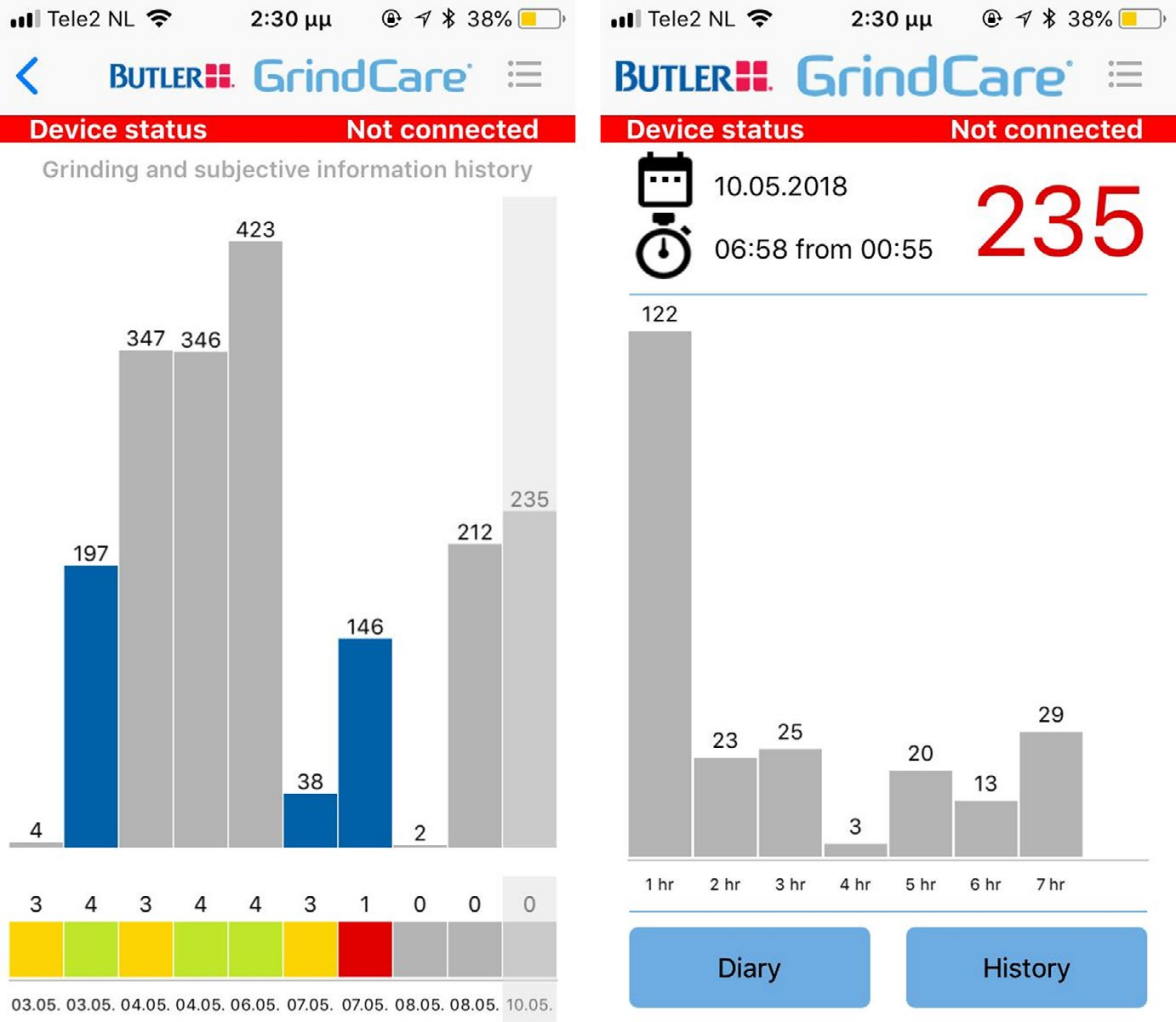
Screenshots of the GrindCare^®^ smartphone app, left: visualisation of the frequency of masticatory muscle events for 10 recordings: each bar represents one recording and right: visualisation of the frequency of masticatory muscle events for one recording: each bar represents one hour of recording

The BUTLER^®^ GrindCare^®^ is a commercially available, CE‐marked, wireless, single‐channel EMG device with dual utility, that is, to monitor masticatory muscle activity, and issue contingent electrical impulses aimed to lower this activity.^18^ It consists of a galvanic tripolar electrode that attaches to the skin over the anterior part of the temporalis muscle with a gel pad (Figure 1). The electrode carries a sensor which registers EMG activity and can issue the electrical impulses. A built‐in algorithm analyzes the EMG signal and scores events based on EMG background noise level.^18^ The validity of the scoring algorithm has been tested against PSG recordings,^9^ suggesting that a single‐channel EMG recording utilising this algorithm can be a good alternative to PSG for the instrumental assessment of sleep bruxism in clinical practice. Scored event data are stored within the sensor. The device's charger is embedded in a separate docking station. Once the sensor is placed in the docking station, data are transferred and stored in the docking station, and deleted from the sensor.^18^ The user can instal a smartphone app which pairs with the docking station through Bluetooth technology. Subsequently, event data are transferred to, and directly visible on the user's smartphone^18^ (Figure 2). The purpose of the app is twofold. First, visualisation of event data on the smartphone app allows the user to directly see how much jaw muscle activity the device detected during each recording. This feature is not obligatory, and participants were left free to choose whether or not they wanted to make use of it. Second, it allows for transferring recording data to a database. The latter was not used for the purpose of the present study. The language of the app is in English.

### 2.4 Outcome measures

Time in the clinic for providing instructions was assessed with a stopwatch. Instructions were given by one investigator (MT) and included an explanation of all device components, instructions for skin cleaning, basic features of the app and transferring data from the sensor to the app. Electrode placement was practiced in front of a mirror.

The diary consisted of two sections that covered 11 domains of interest (Table 1). These domains were chosen based on experience of our research group with the use of a previous research prototype version of the device.^19^ The first section consisted of two parts, one for each evening prior to using the device, and one for each morning after usage. The second section included questions on the overall experience and was filled in at the end of the follow‐up period.

**Table 1:** Structure of diary

	Domains		Method of data collection	Time point
Diary Section 1	1	Feelings and thoughts prior to sleeping with the device	Free text and 5‐point Likert scales,	Evening (D1‐D7)
	2	Time needed to place the device	Minutes and seconds	
	3	Reason(s) for not using the device	Multiple choice with option of free text	Evening (D1‐D7, in case of non‐use)
	4	Degree of disturbance of sleep due to the device	5‐point Likert scale	Morning (D1‐D7)
	5	Experiences related to sleeping with the device	Free text	
Diary Section 2	6	Degree to which sleeping with the device is pleasant or annoying	5‐point Likert scale	End of follow‐up
	7	Ease of using the device components: gel pads, charger, etc	5‐point Likert scale	
	8	Difficulties encountered while using the device	Free text	
	9	Reasons for (not) using the app		
	10	Suggestions and/or complaints regarding the use of the device		
	11	Willingness to recommend use of the device for diagnostic purposes of sleep bruxism	5‐point Likert scale	

Abbreviation: D, day.

In section 1, open‐ended questions invited participants to express their feelings and thoughts on the use of the device before sleep, and, afterwards, their experience with sleeping with it. Additionally, several topics were assessed through 5‐point Likert scales, that is, ease or difficulty in placing the device on the skin (0 = extremely difficult, 5 = as easy as can be), feeling of comfort in the prospect of sleeping with the device (0 = extremely uncomfortable, 5 = as comfortable as can be) and the degree to which the device was disturbing during sleep (0 = extremely disturbing, 5 = not disturbing at all). Furthermore, participants were asked to record the time it took placing device, from the moment of unpacking the gel pad, until the device was placed on the skin. Participants were asked to record any reason for not using the device.

In section 2, open‐ended questions were used to address any encountered troubles, reasons for using or not using the app and complaints and/or suggestions regarding the use of the device and app. Five‐point Likert scales were used to assess how pleasant or annoying it was to sleep with the device, how easy or difficult it was to use the various components, such as gel pads and docking station, and if the participant would recommend the use of the device to others for diagnostic purposes.

### 2.5 Data analysis and final sample size

For section 1 of the diary, deductive content analysis of qualitative data was performed in successive steps, which were adapted from the framework‐based approach, as described by Ritchie and Lewis,^20^ and Pope.^21^ The analysis focused on detecting factors that would facilitate and/or hamper device utilisation, and, to this end, positive and negative experiences prior to, and after sleeping with the device, were identified. First, a chart was created in Microsoft Excel 2010 software. Original textual data on each domain (as described in Table 1) were inserted in the first column by one investigator (MT). They were investigated for initial themes, which were inserted in the second column of the chart. From there, per domain, initial themes were grouped based on conceptual relevance. Extraction and grouping of initial themes were repeated by a second investigator (MV) independently of the first. The two analyses were compared, and the final content of each main theme was based on consensus between both investigators. Inclusion of participants continued until no new themes arose from the diaries, that is, until saturation of data.^20^, ^21^ Saturation was confirmed by including one more participant. Consequently, the final sample consisted of 15 participants. Frequencies of reasons for not using the device were calculated. Textual data of the diary's section 2 were grouped according to relevance. For quantitative variables, descriptive data were calculated using Microsoft Excel 2010 software.

## 3 RESULTS

### 3.1 Sample and recordings

Thirty potential participants were approached between April 2018 and March 2019, and 15 were included (10 female, mean age (SD): 46.7 (16.3)). Reasons for non‐inclusion were as follows: could not be reached after initial screening (n = 3), time limitation/distance from residence (n = 2), no further appointments at ACTA (n = 3), ASA score changed to 3 after initial screening (n = 1) and termination of inclusion due to saturation of data (n = 6). The 15 participants were given the device for a maximum of seven nights, thus, in total, 105 recordings could have been performed. The actual number of performed recordings was 63 (median (25th‐75th quartile): 5 (3‐5.5), range 0‐7). Reasons for not performing a recording are presented below.

### 3.2 Time investment

The mean (SD) time for providing instructions in the clinic was 10.2 (3.2) minutes. The mean (SD) time spent for placement of the device at home was 1.89 (1.3) minutes per recording.

### 3.3 Experiences prior to sleeping with the device

#### 3.3.1 Feelings and thoughts on the use of the device

A median (25th‐75th quartile) of 4 (4‐5) was found for the question on ease or difficulty in placing the device, and 4 (3.75‐4) for the question on how comfortable it feels to go to sleep with the device (Table 2).

**Table 2:** Overview of diary data collected by 5‐point Likert scales

	Questions	Median (25th‐75th quartile)
Diary Section 1	Ease or difficulty in placing the device (0 = extremely difficult, 5 = as easy as can be)	4 (4‐5)
	Feeling of comfort in the prospect of sleeping with the device (0 = extremely uncomfortable, 5 = as comfortable as can be)	4 (3.75‐4)
	Degree to which the device was disturbing during sleep (0 = extremely disturbing, 5 = not disturbing at all)	4 (3‐5)
Diary Section 2	Degree to which sleeping with the device is pleasant or annoying (0 = extremely annoying, 5 = as pleasant as can be)	4 (3.25‐5)
	Ease or difficulty in using the device components: gel pads, charger, etc (0 = extremely difficult, 5 = as easy as can be)	4 (3.25‐5)
	Willingness to recommend use of the device for diagnostic purposes (0 = absolutely not, 5 = absolutely yes)	4 (4‐4)

Analysis of free text data showed that all participants reported a mixture of positive and negative experiences. Most prominent positive experiences included feelings of curiosity and enthusiasm about using the device. These feelings arose from the desire for gaining an insight into one's muscle activity. Satisfaction and surprise about the device's ease of use were reported, as did a sense of comfort after attaching it to the skin. Furthermore, a relaxed, neutral, “neither positive nor negative” attitude was reported, as well as a sense of familiarity after the first day of usage.

Negative experiences included feelings of frustration, disappointment, uncertainty, anxiousness and reluctance. Most prominent negative experiences involved frustration and disappointment regarding detachment of the device during sleep and failure to establish a connection between the docking station and the app. Frustration was also reported about encountered skin irritation, headache, dizziness and the physical interference of the device with wearing glasses. Uncertainty was expressed about whether the device is used in the correct way, whether it will work properly, and whether it will stay attached all night. Furthermore, worrying that the skin will get irritated, sleep quality will be affected, and that it will take too much time to take the device off in the morning were reported. These worries were expressed together with a reluctance in using the device.

It was clear from the diary data that when difficulties in the use of the device were encountered, for example, detachments during sleep, skin irritation or failure to connect the docking station with the app, negative experiences were more prominently expressed.

#### 3.3.2 Reasons for not using the device

Reasons for not using the device were reported for 31 out of 42 unperformed recordings (Table 3). Most frequent reasons reported among participants were not feeling like using the device, malfunction of the device and not sleeping at home. Only one participant did not perform any recordings, and this was due to a general dissatisfaction with the regular clinical care she received (indicated as “don't feel like it”).

**Table 3:** Reasons for not performing a recording, n = 15, multiple reasons were given by some participants

Reason	Number of recordings	Number of participants
Reason not provided	11	4
Skin irritation	7	2
Did not feel like it	6	4
Device or app didn't work	6	3
Not sleeping at home	4	3
Forgot	3	2
Device disturbed children's sleep^a^	2	1
Not knowing how to place the device	1	1
Too tired in the evening	1	1
Time issues in the morning	1	1
Afraid it will disturb sleep	1	1

Note

When the children saw the device attached to his face they tended to play with it, which kept them from their sleep.

^a^ This participant had to attend to his infant children during the night.

### 3.4 Experiences after sleeping with the device

A median (25th‐75th quartile) of 4 (3‐5) was found for the question on the degree to which the device was disturbing during sleep (Table 2).

Analysis of free text data showed both positive and negative experiences. One participant expressed only a positive experience, plainly describing it as “fine.” Another participant expressed only a negative experience, due to the occurrence of skin irritation. All other participants described a mixture of positive and negative experiences. Overall, these participants reported no or minimal bother by the device during sleep. The most important reason for sleep disturbance was detachment of the device (eight participants/13 recordings). Other sources of disturbance were sleeping on the side of the device, skin irritation, awareness of the device's presence on the skin and electrical pulses. The latter occurred in a single participant, who had voluntarily turned them on without proper instruction for this function, due to curiosity.

### 3.5 General experience

#### 3.5.1 Difficulties encountered while using the device

A median (25th‐75th quartile) of 4 (3.25‐5) was found on both the questions of how pleasant or annoying it was to sleep with the device, as well as how easy or difficult it was to use the various components (Table 2).

In this section of the diary participants largely repeated, the issues they had reported in section 1. In addition to what has been described above, one participant reported not being able to place the device in the docking station, one reported annoyance due to hair getting stuck between the device and the skin, and two participants reported that the device had broken. This was confirmed by the investigator (MT). In the first case, the electrode was torn into two pieces when the participant attempted to remove the gel pad. In the second, the device failed to turn on, for unknown reason. Moreover, two participants reported difficulty in using the gel pads. One found that removing them was too time consuming in the morning, and difficult in the evening, due to increased stiffness when they dry out. The other reported placing the gel pads wrongly, thus having to repeat the procedure. Furthermore, difficulty in establishing a connection between the app and docking station, and subsequent failure to gain insight into collected data were reported. This matter was verbally discussed between participants and the investigator when the device was returned. One participant indicated having difficulty with the language of the app being English, and not the native, that is Dutch. The other participants did not report any language issues. Upon receiving the devices, an attempt was made to pair the participants’ smartphones to the docking station together with the investigator (MT). These attempts were all successful.

#### 3.5.2 Reasons for (not) using the app

All but one participants attempted to use the app. The participant who did not use the app used the device for a single night and did not report a reason for not using the app. Insight into the amount of muscle activity was the most prominent reason for using the app. Two other reasons were reported, that is, to check whether the device is working correctly and for contributing to the success of the study.

#### 3.5.3 Suggestions and/or complaints regarding the use of the device

A median (25th‐75th quartile) of 4 (4‐4) was found for the question on whether the participant would recommend the use of the device to others for diagnostic purposes (Table 2).

Some improvement suggestions were made. Alternating between sides of the face, as to avoid irritation of the same spot, was proposed for preventing skin irritation. Recommendations for the app were given: it should show if the stimulation mode is accidentally turned on, if the device is working properly, it should be translated in the native language and have an effective troubleshooting section. Regarding the other device components, it was suggested to provide the docking station with a switch that can disconnect it from the electricity network, since leaving it connected all day felt unsafe. Finally, it was suggested providing the gel pads with grip tabs for easy removal, and that the device should be made compatible with wearing glasses.

## 4 DISCUSSION

### 4.1 Main findings

This study explored the experience of individuals with the use of a portable EMG device (BUTLER^®^ GrindCare^®^) for the assessment of masticatory muscle activity during sleep, in order to detect factors that could facilitate and/or hamper its utilisation.

The median (25th‐75th quartile) number of overnight recordings was 5 (3‐5.5). This shows a good compliance, given that participants were encouraged, but not obliged, to use the device for as many nights as possible during one week. Mean (SD) time investment was low, that is 10.2 (3.2) minutes for providing instructions in the clinic, and 1.89 (1.3) minutes per recording at the home setting.

An overall good experience, with a median of 4 (on a 5‐point Likert scale), was found for the domains of ease or difficulty in placing the device, feeling of comfort in the prospect of sleeping with the device, degree to which the device was disturbing during sleep, degree to which sleeping with the device is pleasant or annoying, ease or difficulty in using the device components and willingness to recommend use of the device for diagnostic purposes.

Qualitative diary data gave more in‐depth information on participant experiences. The desire to gain insight into one's masticatory muscle activity came with feelings of curiosity and enthusiasm, and formed the main motivation to use the device and app. Moreover, satisfaction and surprise about the device's ease of use were reported. These positive experiences were counteracted by negative ones, the most prominent being frustration and disappointment following detachment of the device during sleep and failure to connect the docking station with the app. Furthermore, negative experiences arose from skin irritation and occurrence of headache in a limited number of participants.

### 4.2 Factors that hamper device utilisation

Detachment of the device during sleep occurred in 13 out of 63 (20.6%) recordings. Detachments have been reported in other studies.^11^, ^13^, ^14^, ^16^, ^22^ Takaoka et al^11^ encountered lack of adhesiveness of the EMG device (GrindCare 3.0, Medotech A/S) in one out of 106 (0.9%) participants performing three recordings. Shedden Mora et al reported loosened electrodes and failure to charge batteries of the EMG devices (basic PTA device, Haynl Elektronik GmbH with silver‐silver‐chloride electrodes; T3402 Triodes, Thought Technology Ltd) in nine out of 117 (7.7%) participants performing three overnight recordings.^16^ Conti et al and Yachida et al reported lost electrodes (GrindCare 3.0, Medotech A/S) without providing exact prevalence figures. In all studies, this led to loss of data. Karakoulaki et al^14^ reported loss of connectivity between the EMG device (BiteStrip, Scientific Laboratory Products) and the skin for three out of 45 (6.7%) participants performing single‐night recordings, which were subsequently repeated. Interestingly, the prevalence of electrode detachments in abovementioned studies is quite lower than in the present, a finding that could be explained by the device's design. The electrode used presently carries the EMG sensor. In the studies of Shedden Mora et al^16^ and Takaoka et al,^11^ the sensor was attached to the electrode through a wire. It is thus possible that the weight and/or volume of the sensor in the current study contributed to detachment from the skin. On the other hand, it might be argued that accidental pulling of the wire might contribute to loosening of an electrode. Karakoulaki et al used an electrode which carried the sensor as well, however, compared with the present study, the sensor was less voluminous, though differences in weight are unknown. Moreover, differences in skin preparation and electrode adhesion, that is with gel pad or pre‐gelled type, might have contributed to the variation in the prevalence of detachments. Overall, these considerations should be taken into account in future developments of the EMG device, for example by investigating which features contribute to good skin adherence, as well as in future scientific studies, for example by standardising skin preparation and estimating sample sizes that take possible data loss into account.

Furthermore, despite careful verbal and written instruction, participants encountered difficulties in establishing a connection between the docking station and the app. The app not being in the native language contributed to this difficulty for one participant. All attempts to establish the connection were successful when the investigator, that is a more experienced user, assisted the procedure at the end of the study. This implies that the cause of the issue could lay at the level of the app's functionality, the feature that is related to its performance, ease of use, etc.^23^ Therefore, further development and adequate quality testing of this feature are suggested.^23^

For a limited number of participants, skin irritation (n = 2) and headache (n = 1) decreased the tolerability of the device. Assessments of skin irritation and sensitisation have been performed for regulatory clearance of the device by relevant authorities,^18^ and instructions to discontinue use if skin irritation occurs are included in the user manual. We were not able to retrieve reports of skin irritation in other studies with EMG recordings of masticatory muscle activity during sleep; however, this has been used as an exclusion criterion in one study.^24^ Skin irritation has been reported to limit the use of gel electrodes to short periods of time,^25^ and it might be hypothesised that the sensitive facial skin might be particularly prone for irritation if gel pads are used for longer time periods. It is suggested that skin conditions that render the skin prone for irritation are considered as exclusion criteria for future use of the device. Furthermore, headache lasting for several hours was experienced by one participant. This participant had voluntarily turned on the electrical pulses for the recording after which the headache occurred and it is plausible that the headache was related to this feature, and not to the diagnostic mode of the device. No similar incident could be retrieved from literature. Experiencing headache was included in the informed consent procedure of the current study, based on incidental reports of increased morning headache related to commercial use of previous versions of the device. Out of precaution, it is suggested that this information be included in informed consent procedures.

Other hampering factors were reported, such as difficulty in removing the gel pad from the electrode and difficulty in simultaneously wearing glasses. It is suggested that these factors are taken into account by the manufacturer when designing future versions of the device.

### 4.3 Factors that facilitate device utilisation

Curiosity for gaining insight into one's masticatory muscle activity during sleep was the most important factor that facilitated the use of the device, and the smartphone app was the means by which this need was met. To our knowledge, no studies have utilised ambulatory EMG devices that pair with apps which are available for participants for the investigation of masticatory muscle activity during sleep. A recent study by Prasad et al used comparable technology for assessing muscle activity during waking hours and concluded that this is a promising tool in the field of awake bruxism research.^26^ In line with this conclusion, the results of the current study suggest that visualising masticatory muscle activity on a smartphone app can be beneficial in the field of sleep bruxism, through engaging and motivating the user to comply with multiple overnight recordings. Moreover, the app may be further developed to indicate proper function of the EMG device, as suggested by our participants. Besides improving user experience, this may facilitate acquisition of good quality EMG data and prevent data loss, which has been encountered previously (eg^11^, ^22^). For instance, the app could show whether the device is switched on and data are actually being registered, and monitor the EMG signal quality, in terms of skin‐electrode contact impedance and signal‐to‐noise ratio.^25^ In the study of Prasad et al, real‐time EMG data were collected,^26^ as opposed to the current investigation, in which data were transferred after the end of the recording. Real‐time data collection has the benefit of direct feedback to the user. However, continuous data emission during a sleeping period is unnecessary, and might even be considered as a threatening health hazard by certain individuals,^27^ thus it is suggested that this feature is available for the first few minutes of the registration only, and subsequently turns off.

Both quantitative and qualitative data suggested that using the device was generally considered simple. The wireless design and small number of components may have contributed to this perception. Moreover, compared with other ambulatory EMG devices (eg^14^, ^28^, ^29^, ^30^), a set‐up procedure for defining thresholds of maximum voluntary contraction (MVC) is not required. This is necessary if scoring of EMG events is based on a % MVC method,^31^ which is not the case for BUTLER^®^ GrindCare^®^.^18^ Difficulties and uncertainty about the correct performance of such procedures have been reported,^11^, ^13^, ^22^, ^32^ which, in certain cases, lead to data loss.^11^ The decision on whether an EMG scoring method should be based on a MVC threshold or times‐noise‐level approach should ideally be based on the criterion of validity^31^; however, participant compliance and adherence to the study protocol should also be taken into account.

### 4.4 Future implementations

A smartphone app utilising the ecological momentary assessment (EMA) method was recently introduced for the study of awake bruxism.^33^, ^34^ This too, seems a promising tool for future awake bruxism research.^34^ Studies utilising instrumental methods for assessing both circadian manifestations of bruxism, that is awake and sleep bruxism, are highly needed.^1^ As in other healthcare fields,^35^ smartphone‐based technologies could prove useful. Future studies may aim at developing a multimodal instrument, able to assess both awake and sleep bruxism. An example is an app allowing assessment of awake bruxism by means of EMA, and which can be paired with an EMG device for recording muscle activity related to awake and sleep bruxism.

Furthermore, a note regarding the validity of the EMG device used in the current study should be made. Validity testing of the EMG device's algorithm was done by comparing it to the golden standard of scoring rhythmic masticatory muscle activity on an EMG signal that was acquired during a highly controlled PSG study.^9^ It is possible that the quality of the signal that is acquired by the current EMG device differs from that acquired by PSG, due to differences in, for example, skin‐electrode contact impedance, signal sampling rate and filtering.^9^ This might have consequences for the validity of subsequent scoring of the signal. Thus, it is suggested that future studies additionally investigate the validity of the scoring method of the current device, while taking into account the influence of the signal acquisition method, that is, portable EMG vs full PSG, on the quality of the EMG signal.

### 4.5 Strengths and limitations

The mixed methods design is considered a strength of this study. Quantitative measurements showed an overall good experience with the device, while qualitative data allowed an in‐depth view of the factors that contributed to this good experience, but also to those that prevented it from being excellent. It could be argued that other qualitative methods, for example semi‐structured interviews,^36^ could provide more detailed information. However, this was deemed unnecessary, given that the aim was the investigation of user experience, rather than the construct of a theory to understand health behaviours.^36^ Moreover, by daily diary completion the risk of recall bias was lowered.

Certain limitations are acknowledged. The study sample was selected in a referral clinic, and possibly the experience of users might be different if they were recruited in other settings, for example a general dental practice. In this context, it should be noted that the assessment of bruxism can be important in paediatric,^37^ and certain vulnerable populations, for example those suffering from Parkinson's disease,^38^ or individuals with developmental disabilities.^39^ It is expected that user experiences in these populations can differ significantly from the present. Furthermore, our results were not controlled for the influence of psychosocial and sleep variables, which, to some extent, may contribute to the way one experiences the use of a device they should sleep with.

## 5 CONCLUSION

The use of the wireless BUTLER^®^ GrindCare^®^ device was well accepted for multiple overnight recordings of masticatory muscle activity during sleep. Curiosity for gaining insight into one's muscle activity was the most important factor that facilitated the use of the device, and this need was met through using a smartphone app. Detachment of the device and difficulties in using the app were the main factors that hampered its use.

## CONFLICT OF INTERESTS

M. Thymi reports grants from Sunstar Suisse SA, during the conduct of the study. MC Verhoeff reports Grants from Nederlandse Vereniging voor Gnathologie en Prothetische Tandheelkunde (NVGPT), grants from Nederlandse Wetenschappelijke Vereniging voor Tandheelkunde (NWVT), grants from Stichting Mondzorg en Parkinson, outside the submitted work. Dr CM Visscher has nothing to disclose. Dr Lobbezoo reports grants and other from Sunstar Suisse SA, during the conduct of the study. Grants from Somnomed‐Goedegebuure, grants from Airway Management, outside the submitted work.

## AUTHOR CONTRIBUTIONS

M. Thymi contributed to study conception and design, collection and interpretation of data, and drafting and critically revising the manuscript. Dr F. Lobbezoo and Dr CM Visscher contributed to study conception and design, supervised data collection, and contributed to interpretation of data and drafting and critically revising the manuscript. MC Verhoeff contributed to interpretation of data, and drafting and critically revising the manuscript.
